# Cracks in the pattern: Gallagher’s theory of the self and the dynamics of schizophrenic selfhood

**DOI:** 10.3389/fpsyt.2025.1647545

**Published:** 2025-10-03

**Authors:** István Fazakas, Thomas Fuchs, Samuel Thoma, Cassandre Bois, Tudi Gozé

**Affiliations:** ^1^ Institut für Transzendentalphilosophie und Phänomenologie, Bergische Universität Wuppertal, Wuppertal, Germany; ^2^ Phänomenologische Psychopathologie und Psychotherapie, Klinik für Allgemeine Psychiatrie Zentrum für Psychosoziale Medizin, Universitätsklinikum Heidelberg, Heidelberg, Germany; ^3^ Department for Psychiatry and Psychotherapy, Brandenburg Medical School, Immanuel Klinik Rüdersdorf, Rüdersdorf, Germany; ^4^ Département de Psychologie, Laboratoire Noctua, Université du Québec à Montréal, Montréal, QC, Canada; ^5^ Laboratoire Cliniques Psychopathologique et Interculturelle (EA4591), Université Toulouse—Jean Jaurès, Toulouse, France; ^6^ Service de Psychiatrie, Psychothérapies et Art-thérapie, Centre Hospitalier Universitaire de Toulouse, Toulouse, France; ^7^ Équipe de Recherche sur les Rationalités Philosophiques et les Savoirs (EA3051), Université de Toulouse, Toulouse, France

**Keywords:** selfhood, phenomenology, schizophrenia, mental health, recovery, pattern theory of self, architectonics

## Abstract

The contemporary debate on selfhood in philosophy, psychiatry, and cognitive science unfolds most often between two opposing tendencies: reductionism, seeking to identify a fundamental and minimal core of selfhood, and eliminativism, denying the existence of the self. Shaun Gallagher’s Pattern Theory of the Self (PTS) has emerged as a promising alternative approach. By defining the self as a dynamical gestalt composed of heterogeneous elements and a multiplicity of interacting processes—including embodied, affective, cognitive, narrative, and social dimensions—the PTS offers an integrative and pluralist account of the self. Nevertheless, while the PTS succeeds in avoiding essentialism and supporting pluralism it faces the challenge of providing concrete tools to analyze the organization of self-patterns, particularly in pathological cases such as schizophrenia. We propose a reframing of PTS through the concept of architectonics, rooted in Kantian philosophy and developed in the phenomenological tradition. The architectonic view allows for a stratified and genetic account of self-patterns, attentive to the polyrhythmic unfolding of lived experience across multiple temporal and structural layers. Furthermore, it enables a fine-grained analysis of pathologies and recovery of the self, notably in schizophrenia spectrum disorders, where selfhood appears to be fractured.

## 1 Introduction

The notions of the self, selfhood, and ipseity have gained a central role in contemporary research in philosophy and psychiatry. This conceptual spectrum has been mobilized to bridge theoretical philosophy (primarily phenomenology), cognitive neuroscience, and clinical psychiatry. This is evidenced by highly innovative and inspiring work across these disciplinary fields, particularly in the study of Schizophrenia Spectrum Disorders (SSDs) (see, for example, 1–3).

If the concept of selfhood, or ipseity^1^, is employed by various scholars in ways that are sometimes confusing, these conceptual ambiguities may not necessarily stem from a lack of rigor but rather from the very nature of the object they seek to describe. Paul Ricœur has already pointed out the dizzying polysemy inherent in any attempt to think about the self. He referred to this as the “polysemy of selfhood” (4, 318). This polysemy has more recently been catalogued by Galen Strawson, who provides an often-quoted list of different ways of speaking of the self:

“[T]he cognitive self, the conceptual self, the contextualized self, the core self, the dialogic self, the ecological self, the embodied self, the emergent self, the empirical self, the existential self, the extended self, the fictional self, the full-grown self, the interpersonal self, the material self, the narrative self, the philosophical self, the physical self, the private self, the representational self, the rock bottom essential self, the semiotic self, the social self, the transparent self, and the verbal self.” (5, 100; also quoted by 6, 5).

This list has already been non-exhaustive back then, and in the meantime, there have been other qualifiers that have popped up in debates, multiplying the variations of selfhood.

A first possible reaction to the polysemy of selfhood is to reduce the meaning of the self to one of its aspects, considered a basic, essential feature of selfhood. There would be a fundamental way of being a self, which would ground all other aspects of selfhood and be their condition of possibility. We can find such reductionist tendencies in phenomenology. Indeed, when a phenomenologist finds themself confronted with a list of different layers or registers of selfhood, they can operate a phenomenological reduction of this multiplicity to an essential feature of what-it-is-like to be a self. The very notion of a “minimal self” in contemporary phenomenological literature is a product of such an essentialist (or *eidetic*) reduction—as was the idea of a pure transcendental I in Husserlian phenomenology^2^. As Dan Zahavi puts it, “[r]oughly speaking, the idea is that subjectivity is a built-in feature of experiential life. Experiential episodes are neither unconscious nor anonymous; rather, they necessarily come with first-personal givenness or perspectival ownership. The what-it-is-likeness of experience is essentially a what-it-is-like-for-me-ness” (8, 194). The minimal self is an essential feature of every experience that manifests itself by the first-personal givenness. Understood as for-me-ness^3^, the minimal self is thus the most basic and prereflective feature of selfhood, and it is always implied in higher-level self-experiences. Narrative identity is, for example, always given in a first-person perspective, even when these narratives are made up by others. However, the spectre of essentialism (even in its minimal eidetic form) that haunts this kind of phenomenological reduction has been increasingly criticized in recent years (see, for example, 12). The critics of the minimal self hypothesis, related to mental health, insist that this essentialist or minimalist definition of the self is unable to account for concrete experiences lived by people with SSDs, let alone those with Borderline Personality Disorder (BPD), which involves higher levels of selfhood (13). On the one hand, if the minimal self is supposed to remain present in both normal and pathological experiences, it becomes a difficult challenge to account for altered self-experiences by staying at this “thin” minimal level (14–16). Either the minimal self can be essentially reduced to the formal for-me-ness but then it cannot be disturbed, or the minimal self is not so minimal after all, opening up the question of tracing its limits (11). On the other hand, most of the pathologies that fall outside of the schizophrenia spectrum do need to work with the idea of other layers of the self. Keeping the polysemy seems then to be essential to think about selfhood in concrete situations and calls for a pluralist integrative approach.

In reaction to the polysemy of selfhood, one can also endorse the no-self alternative. The idea behind this option is that we don’t *need* the notion of the self to speak about all the dimensions that are attributed to selfhood. Instead of speaking of a narrative, cognitive, embodied, and other kinds of selves, we can simply speak of narrativity, cognition, embodiment, etc., without a self. Other authors have also raised a more critical question about the very *existence* of something like the self. We can find four major claims for the “no-self” alternative, presented by Thomas Metzinger (17): 1) The ontological anti-realism states that there *is* no such thing as a self; the self is not a real entity. 2) The epistemic argument states that we cannot have any knowledge about selfhood. 3) The methodological anti-realism states that we do not have any methodological constraints to assume the existence of a self. Finally, 4) semantic anti-realism claims that the term “I” does not refer to anything that would actually exist, and therefore, the notion of the self is also semantically empty. The no-self alternatives are popular in Buddhist studies (18) and in some branches of neuroscience, either negating the existence of a self or positing it as a type of illusion or representational phantasy (on a critical assessment of such theses, see 19).

Whether the self “exists” or not (depending on what kind of ontology we endorse) does not yet say anything about the fact that sometimes we do experience it, and psychopathologies offer an attestation of this experience. Indeed, psychopathological conditions such as schizophrenic or borderline states vividly illustrate how selfhood can be weakened, fragmented, or rigidified. It is true that, for the most part, selfhood remains inconspicuous and nearly ineffable in our ordinary experiential life—but this is a privilege of those who are not afflicted by disorders. As a result, asserting the nonexistence of the self may come across as a *validist* argument, one grounded in the normativity of the so-called *healthy*. An integrative model of human experience in general must be capable of accounting for the various forms of self-experience, even altered or weakened. This implies that we need a model of ipseity that has a certain thickness (not a minimalist one) and that doesn’t invisibilize its experience, at least insofar as it can be altered, fragile, or suffering. From the point of view of lived experience, the real question is not whether the self is an illusion or exists in reality, but *how it is lived* and how it structures consciousness. An illusion can be just as important for structuring lived experience as real things, and some illusions might prove to be as irreducible and resistant as supposedly “real things”. Against the no-self alternative, phenomenology seems to be right to insist on the irreducibility of selfhood when it comes to describing lived experience.

Drawing explicitly on these debates, Shaun Gallagher has recently proposed an alternative to the minimal *vs.* no-self debate in his recent book *The Self and its Disorders*, offering an original approach to selfhood in the landscape of contemporary philosophy and mental health. The book takes up the Pattern Theory of the Self (PTS), a theory Gallagher has advocated for and has been developing over the last years in several articles, some of them co-authored with colleagues (for a list of the papers see 6, xi–xii). Gathering his longstanding thought in a coherent argument on how the PTS could be put to use when it comes to thinking about ordinary and disordered forms of being a self, Gallagher’s main point concerns the necessity of integrating several dimensions of the self.

In this article, we first summarize the pattern theory of the self as presented in *The Self and its Disorders*, by focusing on what it means to define the self as a pattern (sec. 2.) As we will see, the self-pattern is defined by its dynamical structure and the main challenge of the PTS is precisely the mapping of the dynamicity. We will focus on the dynamical structure of self-patterns by taking the example of SSDs understood as self-disorders and the possibility of a self-recovery (sec. 3.). Gallagher’s project has the merit, among others, of integrating the question of psychopathology from the outset within his model of selfhood. In his framework of human experience, the experience of madness is not treated as an exception to the rule, nor does he impose any *a priori* demarcation between the normal and the pathological from his epistemological standpoint. Nevertheless, we will see that the Pattern Theory of the Self also raises challenges when it comes to understanding psychopathological experience. We will propose a phenomenological reframing of the PTS, based on an architectonics of lived experience and of selfhood that can help to push further the analyses of dynamical structures in the self-pattern (sec. 4). The reframing of the PTS in the framework of a phenomenological architectonics allows for a multi-layered and genetic approach to the polyrythmical unfolding of the self that can identify possible structural features of the normal and the pathological.

## 2 The self as a pattern

Gallagher’s thesis is that we can understand and describe the self as a pattern. A pattern is defined as a dynamical form that is made up of heterogeneous elements whose configuration can give the impression of a coherent gestalt. As a first approximation, we can think about a pattern on a wallpaper, like an arabesque, or the style of a painter. Behaviors can also be understood as patterns: we can experience different patterns of gestures in a foreign country. A personality could be defined as a recognizable pattern of emotions, style of behavior, schema of relations, etc. Furthermore, we can also think of emotions in terms of patterns. For example, sadness can be thought of as a pattern within the complexity of multidimensional causality: a pattern emerging from biological processes, bodily reactions like modification in posture or crying, reflective judgements, intersubjective relations (being hurt by someone), and other processes. The pattern-theory of emotions is indeed the main inspiration source for Gallagher’s notions of a self-pattern (6, 12–16). For Gallagher “a pattern is a system of factors or processes that lacks any strictly necessary conditions, but rather consists of several jointly sufficient conditions” (6, 16), and a self-pattern is described as a “dynamical gestalt” (6, 17). The pattern theory is a way of organizing multidimensional relations between different ontological registers, such as biological mechanisms, lived experience, embodied experiences, cognitive processes, or social phenomena. The patterns that emerge from these relations are not simply a sum of discrete elements, nor something that would exist outside of these elements, but they are the configuration of the relations.

How does the pattern theory respond to the reductionist-essentialist and to the eliminationist approaches? Against the reduction of ipseity to one essential aspect of the self (for example minimal for-me-ness in phenomenology), the pattern theory advocates for a pluralist and non-hierarchical view. “Rather than positing a hierarchical relation that treats ipseity (prereflective self-awareness) as more basic, and then working outward or upward to more secondary factors, the pattern theory posits a dynamical gestalt” (6, 102). There is no essential feature of the self, be it eidetic, because this gestalt cannot be reduced to only one layer or register of experience, but ranges from affectivity to social structures, from neurocognitive processes to narrativity. In this sense the self is neither in the brain nor only in the experience of mineness. For Gallagher not only “who we are, or what the self is, is more than the brain” (6, 36) but also “the minimal self and what one calls the sense of mineness could only be an abstraction from a much richer multi-dimensional experience” (6, 25). Against the no-self alternatives the PTS claims that *there is such a thing as a self*, and it makes sense to continue to use this notion: the self is not dissolved in its patterns. And the self is not more essential at one register of the pattern, since “there is no self within a self-pattern. There is no single element that by itself could count as a self. A self, of the sort that you are, and I am, just is a pattern” (6, 16). Consequently, there is “no pre-existing entity, agent or self that operates as the organizer or driver of the self-pattern” (6, 120). The self *is* its patterns; or, in other words, the specific mode of existence of the self is its patterns. Thus, the pattern theory of the self does not get rid of the self but avoids reducing selfhood to only one aspect by positing a pluralist, non-hierarchical, integrative model. The PTS is not just a descriptive tool for articulating the different registers of selfhood, it is also an ontological proposition.

But what makes a pattern specifically a self-pattern (and not an emotion pattern for example)? Gallagher suggests that the answer lies not in a more or less complete list of the factors or elements that can enter the constitution of a self-pattern, but in the dynamical organization. He provides a list of elements of the self-pattern (6, 19–23), but the list has only heuristic purposes and might be subject to amendments. The list includes bodily processes, prereflective experiential processes, affective processes, behavioral/action processes, social/intersubjective processes, cognitive/psychological processes, reflective processes, narrative processes, ecological processes, and normative processes. Some theories of the self might work with more elements, some with fewer, depending also on the context of the investigation. The emphasis lies on the dynamical nature of the pattern: it “is not a static pattern like the wallpaper in your room, or a piece of abstract painting hanging in a museum. It’s more like a piece of music being played by a quartet or orchestra” (6, 58). If we take up this musical metaphor, we can also stress that the pattern is not a pattern *of* something, as if there would be a self that can have different patterns, but just as the “ipseity” of a musical piece is the music itself (and not only the musicians, or only the instruments, or only the listeners), the self-pattern is what constitutes selfhood in the first place. However, just as the music is deployed in several layers (musicians, sheet music, performance, instruments, listeners, the social context of a concert, etc.), a self-pattern dynamically crosses heterogeneous dimensions.

There are no essential elements that make a pattern a self-pattern. Instead, it is the recursive self-organization of the dynamical processes that counts. A self-pattern is specific by the recursivity of its self-organization. This means that the processes and operations that make up the dynamical gestalt that is a self-pattern are repeated from themselves, repeating again and again. This implies the idea of an operational closure, which makes it possible for the pattern to maintain its identity and coherence even in an interaction with its environment and the different elements that constantly enter the pattern for the needs of its functions. In our words, the self-pattern is the dynamical form of repeating the repetition within a coherent-enough style to be lived in.^^4^^

Note that this recursive dynamism also implies that some of the elements or processes of a self-pattern are themselves patterns. For example, emotions are patterns that are constitutive of a self-pattern, or intersubjective relations are patterns that mesh with other patterns to form a self-pattern. One could then ask if the self-organizing feature of the autonomous, autopoietic system that Gallagher is looking for only applies to a unique self-pattern for each individual (the pattern that makes you you, and me me) or if individuals can have several self-patterns, i.e., several autopoietic processes working with different organizational principles. Let us take an example: a kind of behaviour that I have in certain situations, for example, when I am with a member of my family, can be described as a pattern. Furthermore, this pattern may have the same qualifications as a self-pattern: recursively self-organizing, dynamical, self-coherent in the dialectics of stability and instability, adaptable, multi-directional, functioning in the interaction between the individual and the environment, etc. But my selfhood is more than just this self-organizing behavioural pattern: I am more than what I am when I interact with this particular family member. There would thus be a partial self-pattern in my self-pattern as a whole. It might be that my self-pattern is composed of more partial self-patterns. But this would mean that there is not only a multiplicity of processes that constitute a self-pattern but even a multiplicity of selves (partial self-patterns) in one individual self-pattern. The problem is to know where to draw the line in this case. Where does *my* self-pattern start? How are different partial patterns integrated into one global self-pattern, and how do these different patterns acquire the quality of being a self-pattern? The task at hand is to map specific dynamics that operate in the self-pattern.

When it comes to describing the organisation of a pattern, Daniel Dennett, John Haugeland, and Scott Kelso are the main sources and discussion partners. Gallagher advances the idea of a “pattern as an irreducible dynamical gestalt where parts or processes are organized in non-linear dynamical relations across a number of time scales” (6, 37). One important point he insists on is giving up on a hierarchical model where the elements or processes would be constructed one upon the other. The idea of a non-linear dynamical organization allows for the modelling of dynamical processes that are mutually conditioned. The so-called “higher” processes can influence the “lower” ones and modify them, and *vice versa.* However, because of this organization, there is no sense of thinking in terms of higher and lower processes anymore, but rather in terms of “metastability” (6, 47). Accordingly, it is not only the elements in a pattern that are in a non-hierarchical relation, but the pattern itself “is not at a higher level than the processes that constitute it” (6, 50). Furthermore, he insists that the meshing of processes with each other in the pattern “is not a fusion into indistinguishable processes” (6, 53). “Within a self-pattern [ … ] we still need to discriminate affect from narrative from cognitive processes, even if we do not want to think of them as operating on different levels, and even if they are in some way causally integrated or meshed and transformed in the process” (*ibid.*).

However, while trying to be radically open and not to impose any pre-given structures, the PTS faces the following challenge: it must account for different processes and for the *criteria* to distinguish them, as well as for their integration into a whole that is organized in a non-hierarchical way. There have been some concerns about whether the PTS can actually do this. And even if it can, it remains open to debate whether this radical openness *a priori* is crucial for healthcare research and clinical decision making. As Myriam Kyselo noted:

“A pattern approach to the self acknowledges diversity but lacks integration, offering no account of the individual as explanatory whole. This poses more than a philosophical armchair problem because what researchers in cognitive science believe the self to be impacts very practically the way they conduct research, from the choice of methodology in setting up experiments and forming hypotheses, to the interpretation of results. It affects how a medical doctor assesses a person’s state of consciousness and well-being or how a psychologist conceives of pathologies of the self and thus whether she chooses to treat with pharmaceuticals, body therapy or social and dialogical intervention.” (20)

A similar critique from Sanneke de Haan concerns the necessity of accounting for a “potential ordering, hierarchy or structure” of the different elements and processes that constitute a self-pattern, and that are particularly important in mental health care and especially for the very possibility of psychotherapy (21, 5–6). Gallagher takes these two critiques seriously (6, 91, 96–97). He does, however, maintain the idea of a non-hierarchical integrative model and situates the problem on the level of the specific dynamics in play in self-patterns. Accounting for concrete dynamics in self-patterns should be able to account for the individual patterns and even influence decision-making in concrete situations. Gallagher is clear on this point and proposes three ways to study the dynamical relations of self-patterns:

“First, a self-pattern is reflectively reiterated in its narrative component. Second, studies of psychiatric or neurological disorders can help us understand the precise nature of the dynamical relations in a self-pattern, and how they can fail. Third, referencing predictive processing accounts, neurological science can also help to explicate, in a non-reductionist way, the dynamical relations that constitute a self-pattern.” (6, 91)

These three approaches are probably not the only ones, and there may well be other ways of studying dynamics in the pattern—the choice of these three seems to be more a matter of authorial preference than theoretical constraint. The answer to a theoretical thematic problem (what does it mean that the self-pattern is a dynamical gestalt)? is thus a demonstration of the *operative* value of the notion, or, in other words, we understand what a self-pattern really *is* by analyzing *how* it concretely operates in different experiences of selfhood. Let’s follow Gallagher’s proposal and take a look at some examples of dynamic structures of self-patterns.

## 3 Dynamics within the self-pattern: the example of schizophrenia spectrum disorders and recovery

From the three ways of mapping the dynamics in a self-pattern, narrativity and psychopathology play a more important role in the book *Self and its Disorders^^5^^*. Gallagher addresses several pathologies, such as Schizophrenia Spectrum Disorders, Autism Spectrum Disorders (ASD), Borderline Personality Disorder, and more. Using the study of psychiatric conditions to reveal dynamical structures that are hardly traceable otherwise, Gallagher reenacts a gesture of classical phenomenological psychiatry and anthropology (e.g. Maurice Merleau-Ponty, Ludwig Binswanger, Henri Maldiney, and more recently Marc Richir). For Gallagher and these authors, pathological conditions are not considered dysfunctional processes and are not defined negatively by the “lack of something”, but they have their own way of being experienced, their own way of organizing different elements inside the self-pattern. As variations of the dynamical gestalt that is the self, they allow us to understand more precisely the dynamics that constitute a self-pattern. Furthermore, these dynamics are reiterated through narrativity. The experiential testimony of a person living SSDs for example, is not just a transcription of the experience (of hallucination for example) but the linguistic enactment of a pattern that is constitutive of selfhood. As Gallagher puts it: “In the case of psychopathology, self-narratives may provide a forensic measure” of changes in the self-pattern, a kind of “linguistic fingerprint” that not only expresses the alterations but is itself a part of it (6, 105).

Schizophrenia is, without any doubt, one of the touchstones of the PTS because it directly concerns the debates on selfhood. It has been argued that self-disorders in SSDs can tell us about the dynamical nature of subjectivity (22), and Gallagher has also argued that different pathologies can help map the dynamics of the self-pattern. A major issue with theories of SSDs is conceptualizing the experience of recovery. Experiential recovery refers to the lived experience of being recovering, in contrast to functional and objective recovery that insist on social reintegration or the disappearing of symptoms. Recovery is a concept that comes from the survivors and users of mental health care system and advocates for the very possibility of being able to live through and with a pathological condition like schizophrenia (23). Recovery is described by these people and qualitative research as the “reconstruction of an enduring sense of the self as an active and responsible agent” (24).

From a phenomenological point of view, experiential recovery can be understood as regaining a sense of self, although it seems to be eroded or fragilized to the extent of losing the continuity of experience (25). From a theoretical point of view, the problem is to account for the articulation between the what-it-is-like to experience schizophrenia and the what-it-is-like to be recovering. These two experiential registers can evolve independently on several levels, or influence each other. Although Gallagher does not explicitly address the problem of experiential recovery in SSDs, we can use it as a touchstone of the viability of the PTS, especially because it involves different registers of the self. While phenomenological models of SSDs put forward the idea of an anomalous self-experience that concerns a prereflective or minimal self, a growing body of literature, based on qualitative studies and personal accounts, insists on the role of self-narratives when it comes to describing recovery (23, 26–28). The theoretical problem is the following: if the minimal self is related to a prereflective self-experience that precedes any kind of storytelling and narrative framing, and if prereflective self-experience is considered to be a more basic layer of selfhood than narrative identity or the narrative self, how can then narrativity function as a basis for recovery? In other words, how to account for the fact that a “higher” layer of selfhood might have an effect on a more basal level?

We would want to argue that without acknowledging that such an influence is possible, recovery would *per definitionem* be impossible, since self-disorders would persist intact under the narratives. We could say that, in this sense, narration does not consolidate the minimal self, but it can continue to act as a support, alleviating or even camouflaging the alteration of the minimal self. On the other hand, one could also take up a narrative model of self-constitution and argue that there is no such thing as a minimal prereflective self that would precede its narrative elaboration. If the self is constituted in and by narratives, recovery would, of course, be a re-elaboration of the self-narrative. Gallagher addresses this idea by discussing Marya Schechtman’s *The Constitution of Selves* (29). Against the idea that the self would be *constituted* in and through narratives, which would actually be a reductionist thesis, Gallagher argues that narratives are only elements of the self-pattern, among other elements. Thinking about the self in terms of a dynamical gestalt where the different elements interact and co-constitute the pattern that *is* the self, promises a solution to the problem of the interaction and regulation between elements of ipseity such as prereflective self-experience and narrativity. The solution proposed by the PTS implies that self-disorders must be reframed not as disorders of a minimal self, but as disorders of the *relations* between different dimensions of self-experience that are dynamically related in the pattern. Accordingly, recovery would not only be a narrative matter, but something that also happens in other areas of the pattern that both determine and are determined by narrativity. For example, writing down one’s biographical story in a peer support writing group is not merely a superficial patching together of discontinuous fragments from the story of my life. This act of writing—especially when shared with others—can in fact bring a sense of coherence to that story, and give me the feeling that I have been the agent of this life, my own. However, the PTS itself does not seem to help us to go further. Hopefully, more qualitative research on self-recovery, allowing for concrete analyses of the meshing of prereflective experience and self-narratives, will provide us with further insights into this matter.

To be sure, these examples show the promise of Gallagher’s integrative approach and its relevance for guiding research in mental health care, as well as in recovery-oriented psychotherapies. Nevertheless, this orientation remains quite underdetermined. In the examples we have seen, the PTS ultimately allowed nothing more than an emphasis on the fact that *certain* structures are entangled, but the task of finding the key to these entanglements is entirely relegated to *a posteriori* observations. As Gallagher himself notes:

“On the one hand, it’s not enough, of course, to simply say that the self-pattern is a dynamical gestalt, and that the different elements of the self-pattern are dynamically related. On the other hand, this is not something one can determine *a priori*, or simply by adopting a particular theory. Rather, we need precisely the empirical and clinical studies of psychiatric or neurological disorders [ … ] to help specify how something like the dynamical integration of self-pattern can occur, how it is ordered, and how it can be disordered.” (6, 96–97)

We could then rightly ask what we won by endorsing the PTS besides the abstract and completely undetermined idea and *desideratum* of integrating various features of the self. It seems that the ability to describe concrete dynamics and integrations does not, in the end, depend on the PTS itself, but rather on the specific theoretical—and experimental—framework one brings in each time to fill the gaps that the PTS leaves open. But why would we then need the PTS in the first place? In this sense, the concrete examples do not offer a satisfactory response to the objection that the PTS fails to provide concrete organizational structures, and as we will argue, in order to do so reintroducing the idea of a stratification of the pattern might prove useful.

The debate on whether one can recognize *a priori* principles for the structuration of the self recalls Husserl’s critique of experimental psychology in *Philosophy as Rigorous Science*:

“experimental psychology is a method of ascertaining possibly valuable psychophysical facts and regularities, but which, without a systematic science of consciousness that inquires immanently into the psychical, lacks any possibility of deeper understanding and definitive scientific value.” (30, 261/303)

If the mapping of the organization of the elements in the PTS is not simply a new version of experimental psychology (extended, embodied, neuroscientific, social, etc.), ordering facts and describing factual regularities, it has to rely on some dynamical structures of the gestalt itself that one can describe independently of individual cases. Or, in other words, there should be a general architectonics, in Immanuel Kant terms, of the gestalt. In what follows, we propose a reframing of the PTS from the standpoint of a phenomenological *architectonics* in order to dig further in the structures that can organize patterns. We argue that a phenomenological architectonics can give a depth to the PTS by reintroducing the idea of different *layers* but keeping the idea of multi-directional effects between the layers.

## 4 Architectonics and genesis of the self

The notion of an architectonics stems from Kantian transcendental philosophy. It is part of the metatheoretical framework, developed in the *Doctrine of Method* of the *Critique of Pure Reason*. Before Gallagher, Kantian ambition was already to organize different registers of knowledge. Architectonics not only refers to such an organization of knowledge, but to a systematic ordering based on principles, or to the “art of systems” (31, 690/A832=B860). This organization of knowledge is not simply a matter of placing knowledge side by side like a patchwork quilt that grows with the addition of new knowledge. Knowledge is integrated organically, which for Kant means as in a living organism, that does not grow by the addition of organs or limbs, but by the growth of the system according to a central structure and orientation (31, 691/A 833=B 861). The architectonics is the very articulation of this structure. Gallagher’s PTS also has a meta-theoretical ambition, which is clearly formulated: “one can think of PTS as operating like a meta-theory that defines a general schema of possible theories of the self” (6, 17). Indeed, the PTS provides an overarching framework meant to integrate different explanatory levels (neural, prereflective, affective, cognitive, narrative, social, etc.). This gives the PTS a Kantian flavor, yet seemingly without the idea of *a priori* principles that are supposed to organize the different theories. PTS as a meta-theory operates more like a guiding heuristic to bring together theoretical and empirical contributions. Is it possible to remain faithful to the radical openness of the PTS as conceived by Gallagher, while at the same time allowing us to think about the mutual articulations and foundational relationships of the registers of experience, thus giving more orientation, coherence and thickness to the PTS?

The problem is to think together the “harder” ontological claim and the “softer” meta-theoretical claim of the PTS. Gallagher seems to have shifted his ontological position over the last decade. Although in 2013 (74) he only states that the PTS was a metatheory, he now subscribes to an enactivist and integrative kind of self ontology^6^. In his book, he assumes the discrepancy between the epistemological and ontological ambition of his model, however he does not offer clear tools to understand the link between these two levels. The task is to think the link between the articulation of the meta-theory and the articulation of the object of this very theory.

This opens the space for a transcendental phenomenological approach, which not only inherits the meta-theoretical and architectonic concern, but also seeks to ground it on the internal articulation of lived experience (as it is clear, for example, in Fink’s phenomenological reframing of the transcendental doctrine of methods in the *VI Cartesian Meditation*, 32). Instead of renouncing to any organizing principles and instead of grounding the system in formal principles, phenomenological architectonics draws its principles from experience itself, whose structures can be brought out by the *epoche* (i.e. the suspension of the natural attitude and the position of being) and specific reductions. These structures imply a “flexible” architectonics (33, 34), capable of accounting not only for the layered constitution of experience but also for the complex dynamics between and within the layers. Thus, architectonics is not only a meta-theoretical tool, but also an architectonics of experience. As Marc Richir writes:

“Architectonics as a method is therefore an ordering of problems and questions according to our concepts, which are essentially philosophical concepts, even when taken with the shifts that Husserl has subjected them to. Corresponding to this, in principle, must be architectonics as the tectonics of the ‘thing’ (*Sache*) itself.” (35, 207, own translation)

The shift from the meta-theoretical ordering of problems and questions to the immanent tectonics of the thing itself (i.e. lived experience) prepares the ground for a phenomenological reframing of self-patterns that goes beyond the problem of integration and seeks to grasp the self as a continuous, processual coalescence of layers and factors into a concrete unity of experience. Pablo Posada Varela refers to this incessant formation of a concrete individual as a whole as a “concreteness in concrescence” (36), pointing toward a mode of becoming that is neither additive nor merely integrative, but one in which multiplicity is already given in its dynamic growing together.^^7^^

**Figure 1:**
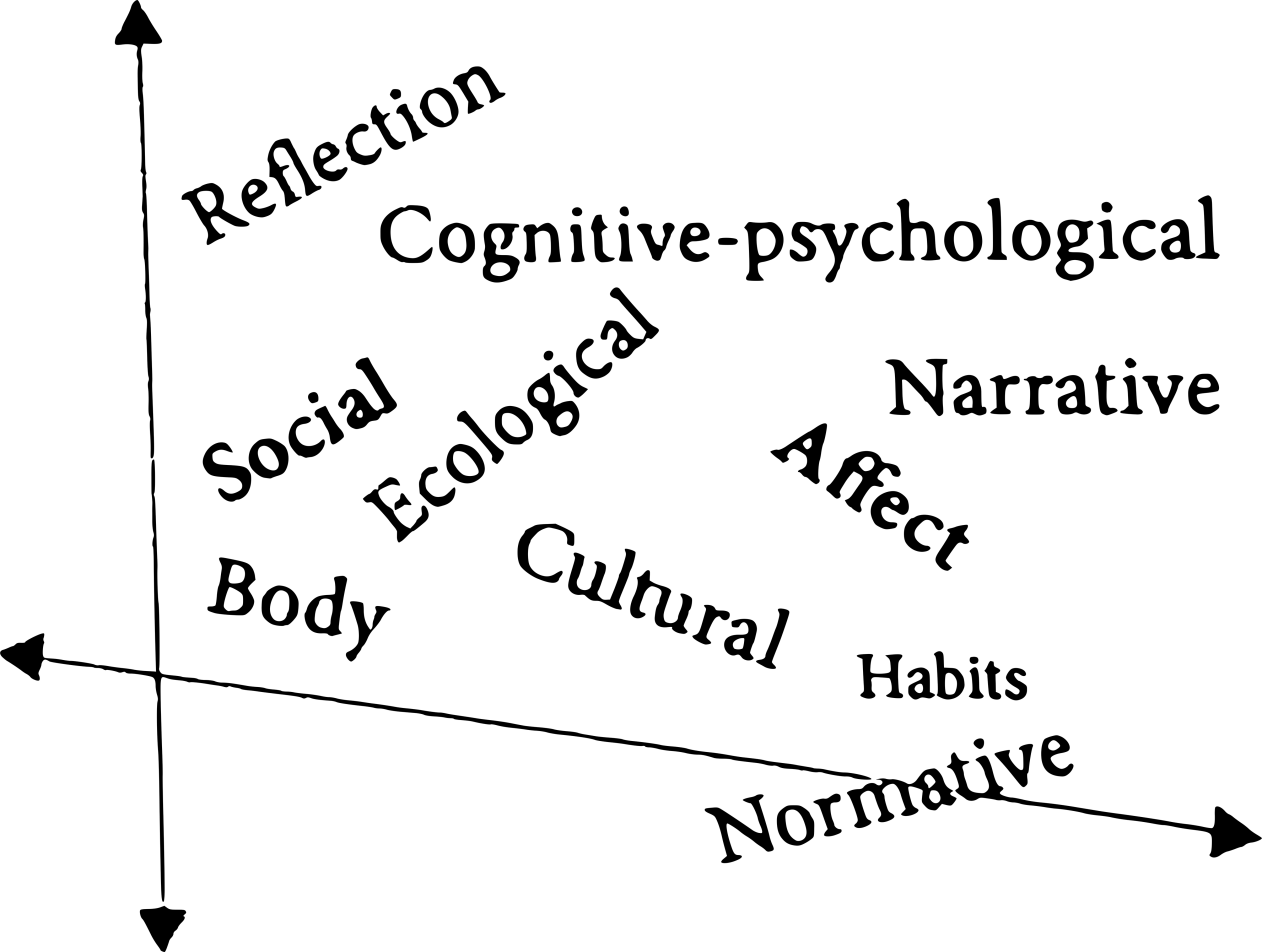
Gallagher’s Cartesian plane as proposed in *The Self and Its Disorders* (6, 75). Source: Copyright Shaun Gallagher (6), reproduced with permission.

The problem of integration is addressed in PTS by the notion of an “enhanced meshed architecture” (6, 68) that is supposed to account for “how different processes and factors integrate or mesh, even if conceiving of the self-pattern as a dynamical gestalt implies a holistic rather than a hierarchical arrangement of factors” (6, 73). While the meshed architecture emphasizes functional integration rather than stratification, it still recognizes the idea of a vertical and a horizontal structuring. Cognitive functions would, for example, be “higher” on the vertical axis than bodily movements, and the horizontal axis includes social and intersubjective structures. Gallagher insists that this is not a hierarchical organization and especially that causality can work in all possible directions. Narratives can, for example, influence motor control, just as movements influence the stories we tell; social structures can influence how feelings are lived, just as behaviour can influence the social construction of identity. We thus have the coordinates for mapping out self-patterns on a Cartesian plane in which all the elements are meshed according to the schema proposed by Gallagher in *The Self and Its Disorders* (**Figure 1**).

Nevertheless, despite this nuanced multidirectionality, what remains strikingly absent is depth, which goes beyond a mere structural ordering towards a temporal unfolding and the genesis of the pattern. Depth, in our view, refers to the generative structuring and sedimentation of experience, introducing a third—intrinsically temporal—dimension to the figure that can account for the relation of transcendental foundation between layers (for example, the body schema has to be grounded to allow narratives to be coherent). That is the genetic production of what we will call an architectonics. Without examining the processes of passive synthesis and sedimentation—which is the project of the so-called genetic and generative phenomenology^8^—the self-pattern remains largely formal despite its dynamics, and its architecture resembles a flattened plane of interactions that are entangled without any genetic relief. Gallagher seems to be aware of the problem; however, he dismisses the idea of a diachronic constitution that operates with levels (6, 54). Although the PTS does work with the idea of different time-scales (6, 55), these scales remain comparable on a Cartesian plane.

The PTS implies placing biological duration, time as it is psychologically lived, and internal transcendental time-consciousness on the same ontological plane. However, these temporal registers are not reducible to one another or to an overarching mono-temporality, nor are they interchangeable. Generative phenomenology—drawing on Husserl’s analyses of genetic and generative constitution—avoids this flattening by seeking to uncover how different modes of temporalization are hierarchically and dynamically nested. These include not only the primal flow of phenomenological time and the passive synthesis of internal time-consciousness, but also the intersubjective and historical dimensions of temporality, such as embodied habits, cultural rhythms, and generational time. Each of these layers has its own structure, its own mode of givenness, and participates in the genesis of a self inhabiting a lifeworld. For example, the nychthemeral rhythm that punctuates lived experience between wakefulness and dreams cannot be compared to the time one needs for sensing the taste of coffee or to the narrated time of meaningful life-episode by reducing them to different time scales in an overarching temporal frame. We propose to take up this idea of multiple temporalizations in the organization of the self-pattern and to push it further in the context of a phenomenological architectonics that shows at the same time how they entail a stratification of experience.

A phenomenological reframing of the PTS allows for investigating this diachronic and multi-directional constitution of the gestalt. Such a reframing entails, nevertheless, two important consequences: 1) the bracketing of the point of view of natural sciences, implying that the different time-scales have to be accounted for in and through lived experience and 2) the reintroduction of the idea of a layered diachrony. The reintroduction of the idea of layers does not entail any impediments to the “meshed” and dynamic nature of the architecture of self-patterns, nor does is lead to a reductionist account by considering the basic layers as dominant, as Gallagher argues (6, 40, 46). Instead, it allows for digging out foundational and constitutive relations, as well as conditions of possibility. The architectonics of phenomenology reveals the layered depth and the relief of lived experience and can account for the becoming of the self across the strata of sedimentations.

Husserl’s idea of a “hierarchical layering” (*Stufenbau*) (39, 218) introduces the idea of an ordering that is not merely functional, but constitutive. In some contexts, Husserl explicitly speaks of a hierarchical layering of genesis (*Stufenbau der Genesis*, 40, 28). Generally, the doctrine of stratification concerns the distinction of different layers of objectivity, world-experience, of apperception, and ultimately of different egological layers (see, for example, 41, 164). We can, accordingly, claim that the self is structured in layers: bodily, affective, habitual, behavioural, prereflective, reflective, etc. These layers are not simply superimposed statically, but they emerge through different genetic processes, such as passive syntheses, kinesthetic sedimentations, temporal constitution, institutions and re-institutions, or processes of idealization. Some layers constitute a basis for the constitution of others. Bodily movement, for example, is the basis for the unfolding of space (42), internal time consciousness is the basis for the experience of continuity and of objective time (43), passive associations are the basis for the formation of experiential types (44), sedimentations of apperceptions and habits are the basis for the formation of personality and life-history (41, 109). Moreover, longer-lasting background mood states tend to favour short-term emotions; these in turn give rise to higher-level images or thoughts, including reflective thoughts, anticipations and narratives about the self. Basic or background experiential states thus exert a constraining or facilitating influence on more foreground states. Conversely, foreground states can stabilize or modify background states; the influence is thus bidirectional or circular. The flexible system of all these different layerings can be seen as the phenomenological architectonics of lived experience.

Since these layers are themselves results of a complex genetic unfolding, their internal articulation is not given as a ready-made system, but it isn’t simply empirical or *ad hoc* either. Their dynamic coherence must be uncovered through specific phenomenological reductions. The most important reduction, in this context, is what Husserl called the dismantling reduction (*Abbau-Reduktion*) (for a historical reconstruction, see 45, 46). Although it might be related to Heidegger’s idea of “destruction” and it has inspired Derridean “deconstruction”, it must not be confused with any of these (46). Instead, the dismantling reduction seeks precisely at peeling back different layers of constitution in their *genetic articulation*. It can concern historical sedimentations in the lifeworld, as well as sedimentations in the history of an individual subject, ranging to the depth of its pre-personal temporal unfolding. The method of *Abbau* is radicalized in Husserl’s late work where he argues that the genetic articulation is not a temporal sequence of *episodes*, but it unravels a stratified simultaneity. As Husserl notes in an important passage of the *C manuscripts:*

“For all *Abbau*-reduction, the fundamental theorem holds that the layers of *Abbau* are not constituted in genesis as such, in a genetic sequence that corresponds to the order of foundation. *While each layer does correspond to a layer in genesis*—since all intentionality through which the pre-given world is constituted is genetically acquired and engaged in an ongoing genetic process—*all geneses of all layers function together immanently in time; they are coexisting geneses.*” (47, 394, own translation and italics)

This rather complicated passage suggests that the layers uncovered by the dismantling reduction do not exist in themselves as such, they are not independently constituted as *discrete*, independent levels. Rather, they operate in a simultaneous genesis where the different strata of constitutions interpenetrate each other with their own temporalities, forming a coherent, polyrhythmic whole. If the layers of genesis *do* correspond to layers of foundation (as Husserl rectifies), they are not independent because they are themselves in a constant co-genesis. The passive syntheses continue to unfold simultaneously to the formation of narratives that make up narrative identity; the genesis of the lived body continues to be in function while kinaesthesia sediments and habits are formed; affectivity is in constant genesis while cognitive processes operate. This polyrhythmic genetic architectonics lays the groundwork for a non-linear and multi-temporal notion of self. It allows precisely for accounting for the depth in the self-pattern, for the superpositions of different genetic temporalities.

This polyrhythmic genetic model aligns with approaches in developmental psychology^9^. We can for example observe how early affective and sensorimotor experiences can shape later cognitive, narrative or reflective structures. These differences are not only on a vertical axis in a cartesian plane, rather they are genetically distinct layers, which structure the architectonics of the self. Once the adult self is formed, these layers are of course co-constitutive of the self-pattern, they function polyrhythmically, but through the dismantling reduction we *can* distinguish them precisely as different *foundational layers*. This distinction remains valid even if we recognize that layers that emerge later in the constitution can have an influence—and sometimes a transformative effect—on the layers on which they were founded. This also implies—and this is a crucial ontological point—that the layers discovered in the dismantling reduction regime do not *exist* as such. They arise precisely through their relations of foundation, and if the delimitation from one to the other is never clear, we can still determine which is foundational and which is founded.

In generative phenomenology, important corresponding analyses can be found in Husserl’s late manuscripts on primordial childhood (*Urkindheit*). In an often quoted manuscript on the *The Child. The First Empathy* (48, 604*sq*) Husserl pushes the dismantling reduction to its furthest limits, arguing that the archaic self is a pre-egoic bodily affection that later is awakened by transcendental intersubjectivity to its primal self-apperception. He then shows how this pre-egological and pre-personal layer functions as the basis for the different sedimentations through which first habits and gestures and later even language are constituted in an intersubjective environment implying a constant interaction with others.

For the architectonics of the self, this implies that in its most archaic dimension, the self can be described as a bodily-affective self, something which we could call a coenesthetic selfhood^10^. Husserl speaks of a pre-egolocial passive reception of a *hyle*, i.e. a bodily affection that is related to the most basic affectivity as a participation to an originary intersubjective life (48, 604). This coenesthetic self is then the basis of the agentive self, that learns to inhabit and control its body (Husserl speaks of an originary “I can” [41, 128, 133, 148–149], or of a “praxis” [48, 607], but we can also think about Maine de Biran’s philosophy of the effort here [56]), which also serves as the basis of the sedimentations of gestures. On the basis of this habitual sedimentation gestures of language arise, giving birth to a symbolic (linguistic and semantic) self or personhood, that once again is at the basis of what will become the narrative identity. The analysis of the genetic unfolding of the self guides the distinction and the articulation of different architectonic layers. And these layers remain in function in the self-pattern, that is in this sense a superimposition of different gestalts, rather than a flat—although dynamical and diachronic—gestalt. Briefly put, a self-pattern always already has its genetic depth, that follows the distinction of architectonic strata. Both phenomenology and developmental psychology converge in viewing the self as temporally *thick* and the architectonics is precisely the art of articulation of the thickness of the self.

**Figure 2:**
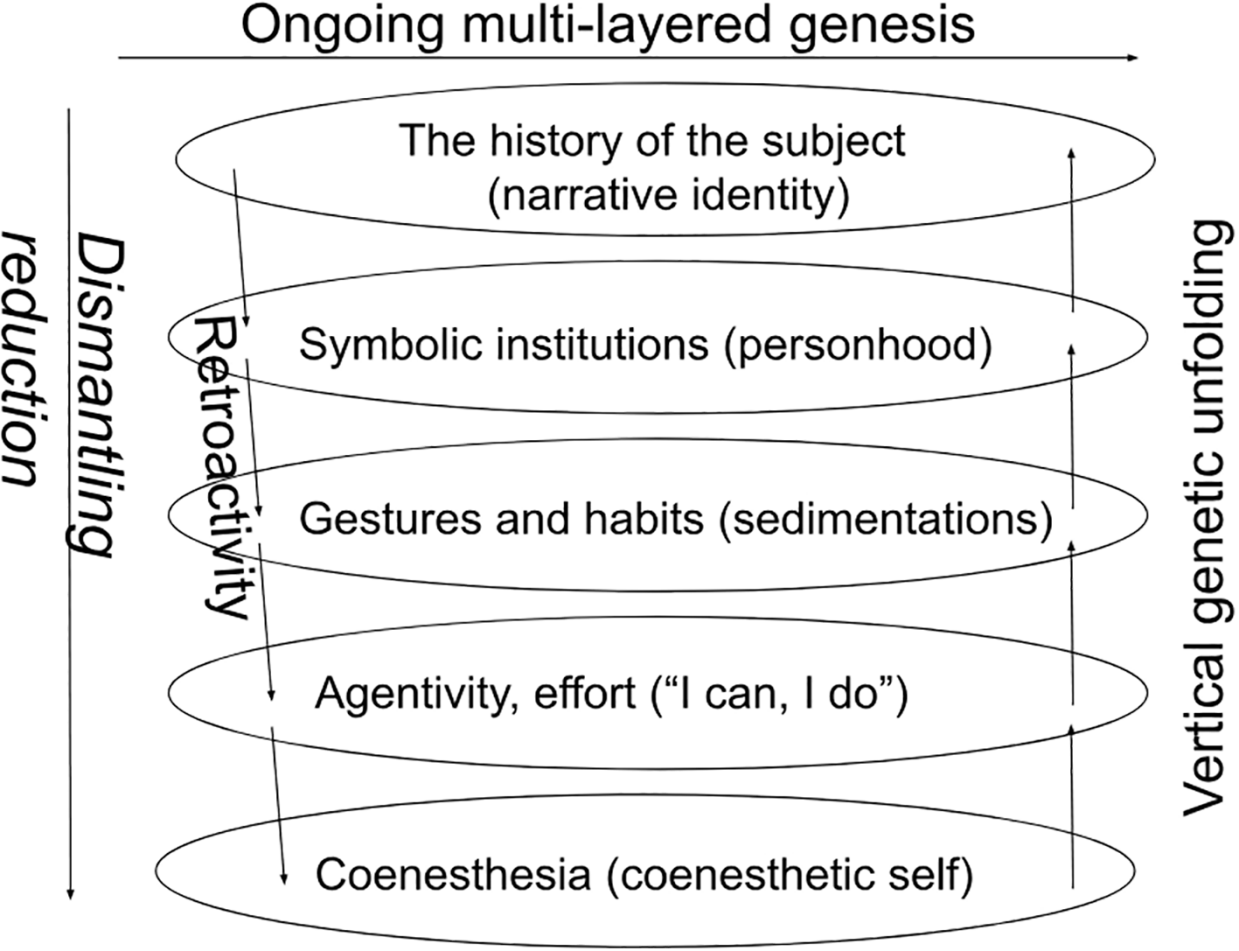
Example of a dismantling reduction.

This move does not discard the PTS, but rather grounds it by offering a layered and genetic model that can account for structures in the pattern that do not depend merely on empirical observation but unearth tectonic regularities in the stratification of experience in general. Of course, Husserl’s genetic phenomenology is open to amendments, and the architectonics that is retrieved by the dismantling reduction can vary according to the conceptual fine-tuning that is needed for the analyses. Nevertheless, some structures of the genetic layering can be seen as binding: for example, the coenesthetic self is necessarily more basal than the narrative self, even if there can be a complex dynamics between the two, ranging from feedback loops to retroactive reframing or even a cleavage (*Spaltung*). Such a phenomenological perspective, tracing the formation of the self(-pattern) back to its pre-egological bodily origins, aligns with the idea of a genetic approach to nested selves put forward in psychoanalysis and neuroscience (57, 58) (**Figure 2**).

The dismantling reduction does not however uncover real *entities* that can be found somewhere (in the brain for example), but only layers of self-experience which will depend on the concrete analyses which are carried out. As an example, this has been done for describing the layering of self-narration in BPD (59–61), imaginative experience in SSDs (62) or the genesis of basic trust in early childhood (63). The architectonic model reintroduces the idea of foundational layers, while renouncing the idea of a static eidetic reduction. Instead of an essentialist (eidetic) reduction, the dismantling reduction uncovers interdependent layers, layers that function simultaneously, although they build one on the other genetically. While the genetic unfolding is bottom-up, the simultaneous polyrhythmic functioning allows for top-down effects, constituting a multi-layered dynamic pattern.

Before coming to our conclusion, it is important to emphasize once more that according to the principle of the dismantling reduction and the idea of a polyrhythmic genesis, the archaic self (the self of the *Urkindheit*) is not just a mere chronological phase, but an ever-present genetic foundation that continues to operate in the background of adult subjectivity as one of its layers. The idea of tectonics of the archaic—taken up from Richir’s and Varela’s definition of the phenomenological architectonics—gains its full importance here. Richir insists, more than Husserl, on the possible dynamics and tensions *between* the layers introducing “movements, overlaps, ruptures, faults, thrusts, etc., of the archaic, whose traces the phenomenological field continuously retains, most often in a way that is paradoxical in relation to Husserl’s calm stratigraphy” (35, 207, own translation). The idea of tensions between the strata is not only a structural insight but has a crucial relevance for psychopathology. The architectonic model makes it possible to describe how disruptions may occur when the rhythms of different layers fall out of sync. For example, coenesthetic bodily affectivity may resurface in ways that interfere with cognitive or narrative processes (described traditionally as cenesthopathies; 64). In some cases, entire “higher” layers—such as linguistic or narrative structures—may become unstable or subducted (Mignard coined the term “subduction mentale morbide”, 65) under the pressure of more primitive, affective strata. Pathological phenomena like depersonalization, fragmentation of narrative identity or affective incoherence may thus be understood as fault-lines in the tectonics of selfhood—zones where the polyrhythmic genesis becomes disarticulated, where sedimented strata crack or fail to co-function. This issue brings us back to the question of the normal and the pathological, on which we propose to conclude.

## 5 Conclusion

As we have seen, *The Self and its Disorders* offers an overview of recent developments in the pattern theory of self. This theory has an ontological thesis on what the self *is*: namely a pattern, a dynamical gestalt and nothing else. Although the gestalt is not defined by the elements that make it up, we can distinguish several processes that might participate in the constitution of a self-pattern. The self-pattern can include bodily, pre-reflective, narrative, cognitive, ethical and other processes. The PTS doesn’t decide *a priori* on a set of elements (although it provides a list of processes that can play a role). It is mostly concerned by the dynamicity of the gestalt which is the very core that makes up the ipseity of a pattern. This dynamicity is rarely analyzed in an abstract and general way. We can find plenty of methodological considerations on how to map this dynamic in a meshed architecture, but these considerations mostly remain negative: it is *not* reductive, it is *not* a hierarchical model, it is *not* exclusive, etc. However, after all these negative determinations, it seems as if we’re left with rather little on the purely theoretical level and that the task of mapping out the possible structures in the patterns is left to empirical observations. This has important consequences when it comes to drawing the line between a normal and a pathological self-pattern or between an experience of well-being and suffering.

The PTS itself, in its current form, doesn’t offer sufficient criteria to distinguish what makes a functional or suffering self. It is indeed not in its scope to provide such criteria, although it is sensible to rigidification in the self-patten, which might be associated with some disturbances. In a recent study, published by Anya Daly and other colleagues of Gallagher, the authors propose to apply the pattern theory of the self to qualitative research (66). They propose a framework for front-loaded phenomenological qualitative interviews, which they call the *Examination of Self Patterns* (ESP). The ESP is designed to tackle the inherent complexity of disturbed self-patterns in psychiatric disorders. However, they make it clear that their “aim is not to come up with necessary and sufficient conditions for a given configuration of the self-pattern to be classified as disordered” (66). On the other hand, they do provide a minimalistic definition of what might be considered a disordered pattern. They indeed state that “many psychiatric disorders can be understood as disruptions in the self-pattern”, and a note explains that “a change in the self-pattern becomes a disturbance when that pattern becomes stuck in a form that contributes to maladaptive function” (66). This suggests that the distinction between the normal and the pathological within the self-pattern concerns its dynamicity. A “stuck” or rigidified self-pattern would be closer to the pathological, a dynamic or moving self-pattern to health. Such a criterion makes sense if we consider that the dynamicity of the gestalt is in the very definition of the self-pattern.

The architectonic reframing of the PTS allows for a deeper investigation of the dynamicity of the pattern and its possible disorders by introducing the idea of a stratified, polyrhythmic dynamics. Such a view can provide some criteria to identify the conditions under which a self-pattern may be called disordered, and it can also distinguish different disorders by situating their origin in the layered architectonics. One can, for example, describe different types of disorders: desynchronization, subduction, or sometimes what we could call an architectonic *catastrophe* (in the etymological sense of *kata* “down” and *strephein* “turn”), a complete reversal of constitutive layers. It is, for example such a reversal of the “empirical time” and the “transcendental time” that Blankenburg observed in his patient, Anne Rau (67). Anne Rau has to produce herself from herself in a way that Blankenburg also describes as the schizophrenic autism, that “generally appears where the empirical I is compelled to take on the task of the transcendental I, to guarantee an ‘*autos*,’ a self” (67, 130). And one can situate the architectonic location of what Minkowski has called a “trouble générateur” (68) in different disorders. It is, for example, common to observe the fragmentation of narrative identity both in borderline personality disorder (59, 61) and in schizophrenia (69, 70), but the “trouble générateur” is not situated on the same architectonic level in both cases.

Last but not least, the architectonics of the self can provide a theoretical framework for investigating recovery in severe mental illnesses. In SSDs, for example, the main question is where to situate recovery, and in which layers we can think about self-recovery in the polyrhythmics of basic self-experience and self-narratives. This would make it possible to understand more precisely how higher-level layers, such as narrativity, can participate in restructuring or sustaining a fragmented or weakened embodied experience. This helps us understand how the so-called *phenomenological compensation* (68, 227–230) works. This concept refers to the compensation of a pre-reflective (or transcendental) function by a higher level process of the self. In this sense, one may indeed exhibit a persistent vulnerability of the basic self throughout life, and yet still manage to live a “healthy” life—because this vulnerability is compensated for by a kind of experiential or even intersubjective patch, prosthesis or scaffolding. If we take from the pattern-approach the idea of a dynamic gestalt in which different elements, registers or dimensions interact and mutually condition each other, and from the phenomenology of schizophrenia the idea that the disturbance lies at a basic level, the architectonic approach, which conceives of the gestalt as a genetic and polyrhythmic stratification, makes it possible to think about the effect of compensation on the basal layer. The point is to consider that compensation is not merely a superficial add-on, but that it can (at least theoretically) have an effect (top-down) on more basic processes. To use a metaphor of W. James, the higher layers might have an effect that allows one to regain, even if only partially or episodically, the “warmth and intimacy” (10, 239) of their experiences (see also 15). Considering this possibility is necessary in order to design empirical research without presupposing from the outset that recovery cannot reach the most basic level of self. On the other hand, this hypothetical possibility does not say anything about a basic vulnerability—different from disorder or disturbance—that might persist in the gestalt, susceptible of making cracks in the pattern.

We arrive thus to a conception of health and recovery grounded in the *multi-layered dynamicity* of the self-pattern. This understanding allows us to describe how, despite the persistence of psychotic symptoms such as hallucinations or delusions, a person may still live meaningfully and adaptively through the process of recovery. To be healthy, then, is to possess a dynamic, plastic, and creative configuration of the self—one capable of navigating and transforming the tensions between stability and instability, closure and openness, continuity and rupture. This conception aligns with the enactivist model proposed by Gallagher, in which health is understood as the coherence of a system in its dialectics of self-regulation and perturbation (6, 61–62), as well as with phenomenological accounts of the dialectic between openness to alterity and the closure necessary for self-constitution (71, 72). Such an architectonic view of the self allows for a non-dualistic understanding of health and illness, in which fragile, even pathological forms of selfhood may nevertheless express a singular way of *living with, through, and beyond illness*.

The phenomenological reframing of the PTS endorses the idea of a pluralist view of the self and the necessity of integrating the different registers of the self in a complex model that works with the idea of a multi-directional constitution. Instead of relegating the task of mapping out the structures of the self-pattern to a posteriori research, it rather takes on the task of describing the stratification of the self through the analysis of its hierarchical layers (*Stufenbau*) and through the dismantling reduction (*Abbau*) of its co-constitutive genetic layers. The exploration of trajectories of embodied (and embedded) self-experience from prodromic or at risk phase to illness and recovery in a dynamic multilayered genesis could enable an explicitation of the dialectics between plasticity and rigidification of self pattern in schizophrenia spectrum disorders. This research direction could be a fruitful complement to the follow-up studies of basic self disorders (3, 73) opening up the attention to the interaction between the layers and even the possible top-down influence on higher layers on the more basic ones. Focusing on the polyrhythmy of different genetic strata allows us to see not only the disorder but also the experiential resources of the person. This is relevant for thinking about recovery dynamics, but also for developing therapeutic strategies that build on these resources rather than merely correcting disorders.

## Data Availability

The original contributions presented in the study are included in the article/supplementary material. Further inquiries can be directed to the corresponding author.

## Footnotes

1 In this paper we use these two terms (selfhood and ipseity) interchangeably since the English *self-hood* and the Latin *ipse-itas* are constructed in exactly the same way. Although in some contemporary texts one of the terms can acquire a more concrete meaning (e.g. ipseity referring to a specific type of selfhood like “minimal” self) we stick—unless specified otherwise—to a general meaning.

2 Husserl indeed speaks sometimes of an “non-personal consciousness”, “without a body” and even “without a soul” (7, 119). The minimal self can be thought of as a contemporary re-effectuation of this type of reduction.

3 It should be noted that *for-me-ness* is not equivalent to *mineness* or *me-ness* (9). Although Zahavi’s philosophical work does not always reduce the self to for-me-ness, recognizing a dimension of mineness or a more “robust” meaning of for-me-ness as the quality of “warmth of intimacy” (10, 239) of self-experience that can be disturbed (11), we can also find a “thinner” reading of for-me-ness in his works (8). It is this latter that we are addressing here.

4 This might seem tautological, but Gallagher immediately dismisses the criticism: self-organizing does not mean “organizing a self”—which indeed would be a circular answer to the question of what makes a pattern a self-pattern–rather it must be understood as a kind of autopoiesis, in the sense of Francisco Varela. (6, 27). More technically Gallagher describes what makes a pattern specifically a self-pattern by drawing mainly on Varela and distinguishing five points: (i) recursive meshwork of constituting processes, (ii) further recursivity in action and habits or adaptability (Di Paolo), (iii) coherence in the dialectics of stability-instability of the system, (iv) reciprocal causality that goes beyond dyadic reciprocity and involves a multi-directional multiplicity, and (v) organism-environment interaction (6, 61–63).

5 Predictive processing is the least developed approach by Gallagher, yet this idea is very fruitful. We will return to this point in footnote no. 8 in section 4 “Architectonics and Genesis of the Self”.

6 We are grateful to Reviewer 2 for pointing out this shift in Gallagher’s thinking.

7 Our proposed reframing of the PTS, based on a transcendental architectonics of experience, is inspired by these methodological contributions of Richir and Varela. Marc Richir (1943–2015) is a Belgian philosopher who has undertaken a refoundation of phenomenology that still remains mostly unknown in the English-speaking community because of the lack of translation. Pablo Posada Varela (1975–2024) is a Spanish philosopher and one of the most prominent continuators of Richir’s work. He has developed a contemporary reading of Husserl’s mereology (2024).

8 Here we refer to the generative constitution of experience as Husserl proposed it in the *The Crisis of European Sciences and Transcendental Phenomenology* (75) and as reframed more recently by Anthony Steinbock (37) and Claudia Serban (38).

9 Gallagher discusses briefly the developmental perspective in the context of his analyses of torture and solitary confinement, but not as a tool for the articulation of the layered genesis in the depth of self-patterns in general (6, 246–247).

10 The notion of coenesthesia, introduced by Hübner and Reil in 1794, refers to an immediately feeling of our bodily existence. It has been also associated to a vital sense (*Vitalsinn*) and has been described as the core of selfhood: “The feeling of self at its lowest level is the common feeling, and this itself is capable of various gradations, so that we can conceive of it, in its embryonic state, as the unity of the bodily mass appearing to itself in the deepest darkness” (49, 699, quoted by 50, 5, own transl.). On the history of the notion, see also 51; for a phenomenological reading, 16, 52; and for an elaboration of the relation between coenesthesia, common sense and schizophrenia, 53). The phenomenological architectonic approach to the coenesthetic dimension of selfhood aligns with contemporary research in cognitive neurosciences on interoception—although they have different epistemological foundations. Indeed, the notions of coenesthesia and interoception are not only historically related; but sometimes even synonymous (54). Contemporary definitions of interoception as the representation of one’s own “bodily milieu” also responsible for the “sense of self” (55) seem to address the same phenomenon. Specifically fruitful in this context is the predictive processing approach, which Gallagher has described as the third major way of mapping out self patterns. The study of the relation between the phenomenology of coenesthesia, the architectonic model of the self, and the patterns of predictive processing in interoception regarding the constitution of levels of ipseity would, however, require much more space and should be the object of future research.
